# Salvage surgery following tyrosine kinase inhibitor treatment for advanced non-small cell lung cancer

**DOI:** 10.1186/s40792-024-01950-6

**Published:** 2024-06-20

**Authors:** Masao Kobayashi, Soichiro Funaki, Hideki Nagata, Mitsugi Furukawa, Eiichi Morii, Yasushi Shintani

**Affiliations:** 1 Department of Pulmonary Medicine, Osaka Saiseikai Senri Hospital, Suita, Japan; 2 Department of General Pathology, Osaka University Hospital, Suita, Japan; 3 Department of General Thoracic Surgery, Osaka University Hospital, 2-15 Yamadaoka, Suita, Osaka 565-0871 Japan

**Keywords:** Lung cancer, Tyrosine kinase inhibitor, Salvage surgery, Epidermal growth factor receptor mutation, Systemic therapy

## Abstract

**Background:**

No standard therapy for non-small lung cancer patients that have acquired resistance to tyrosine kinase inhibitor (TKI) therapy has been established. Some can be effectively treated by salvage surgery, though indications for that procedure remain unclear. Reported here is the clinical course of a patient who experienced early post-operative distant metastases.

**Case presentation:**

A 48-year-old woman without symptoms was referred to another hospital for abnormal chest radiography findings and diagnosed with adenocarcinoma of the left lower lobe (cT2aN3M1b, stage IVB; TNM staging 7th edition). Gene mutation analysis revealed positive for epidermal growth factor receptor exon 19 deletion. Afatinib treatment was started, resulting in partial response, though regrowth of the main tumor was noted 1.5 years later. Bronchoscopic re-biopsy findings revealed a T790M point mutation and afatinib was switched to osimertinib. At 1.5 years following the start of osimertinib administration, the primary tumor was found to have regrown again and stereotactic radiation therapy was administered. Findings at 3.5 years after osimertinib administration indicated that all lymph nodes and distant metastases, excluding the primary tumor, were well controlled, and the patient was referred to our hospital for salvage surgery. Osimertinib was discontinued, and a left lower lobectomy with a left lingular segmentectomy and pleural biopsy were performed. The patient was discharged following an uneventful postoperative course. Three days after discharge, glossodynia developed and examination findings revealed tongue metastasis. The symptoms improved following re-administration of osimertinib, though right adrenal gland metastasis appeared 8 months after surgery. Radiation therapy was performed for tongue and right adrenal gland metastases, and the patient was alive 1 year after salvage surgery without out-of-control lesion appearing after the radiation therapy under the administration of osimertinib.

**Conclusion:**

The present patient experienced multiple instances of systemic recurrence after undergoing salvage surgery. Experience with this case indicates that systemic therapy is essential for patients with distant metastatic lung cancer even following salvage surgery for the primary tumor.

## Background

Lung cancer is the leading cause of cancer mortality throughout the world, accounting for 18% of total cancer deaths [1]. Adenocarcinoma is the most common type and Shi et al. reported that 51.4% of Asian lung adenocarcinoma patients harbored an epidermal growth factor receptor (EGFR) mutation [2]. An established first-line therapy for EGFR-mutated lung cancer cases with distant metastasis is administration of an EGFR tyrosine kinase inhibitor (TKI) [3]. While a T790M point mutation is one of the resistance mechanisms known to limit the effects of first and second generation TKIs, use of osimertinib, a third generation TKI, was found to provide an average 10.1 months of progression-free survival (PFS) in T790M-positive advanced non-small-cell lung cancer cases [4]. However, no standard therapy has been recommended for lung cancer patients who have acquired osimertinib resistance [5].

Salvage surgery for viable tumor tissue following TKI treatment is one of the available therapeutic strategies. Ohtaki et al. [6] and Lin et al. [7] reported favorable outcomes of salvage surgery, and presented findings indicating survival improvement efficacy. Herein, findings and the clinical course of a patient who experienced early post-operative distant metastases are reported.

## Case presentation

**Fig. 1:**
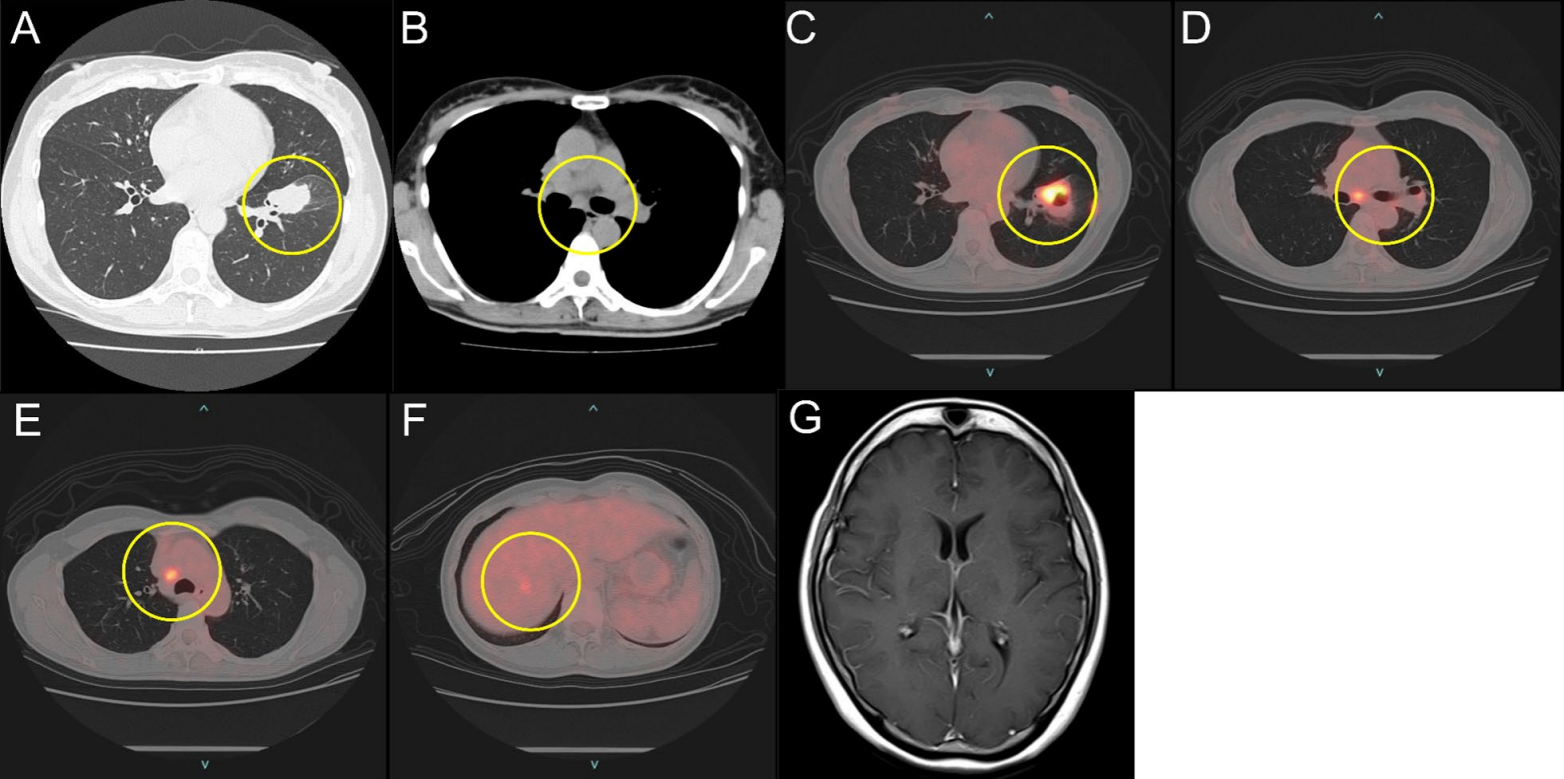
CT images showing **A** 36-mm tumor in left lower lobe and **B** swelling of the subcarinal lymph node. PET-CT revealed **C** a high level of accumulation of the primary tumor (SUVmax 10.5), as well as metastases in the **D** subcarinal lymph node, **E** right mediastinal lymph nodes, and **F** liver. **G** Contrast enhanced brain MRI findings showed no brain metastasis

A 48-year-old woman with a history of asthma and who never smoked was referred to another hospital for abnormal findings in a periodic health examination. Chest radiography revealed a nodule in the left lower lung field, while computed tomography (CT) findings showed a 36-mm tumor in the left lower lobe and swelling of subcarinal lymph node (Fig. 1A, B). A laboratory examination revealed elevation of serum carcinoembryonic antigen at 60.6 ng/ml without other abnormalities. Bronchoscopic biopsy results confirmed a diagnosis of lung adenocarcinoma and gene mutation analysis showed positive for EGFR exon 19 deletion. Findings obtained with positron emission tomography-computed tomography (PET-CT) indicated a high level of accumulation of the primary tumor (SUVmax 10.5), along with metastases in the subcarinal lymph node, right mediastinal lymph nodes, and liver (Fig. 1C–F). Contrast enhanced brain magnetic resonance imaging (MRI) showed no brain metastasis (Fig. 1G). Based on the imaging findings, the tumor was diagnosed as clinical stage IVB adenocarcinoma (cT2aN3M1b; TNM staging 7th edition).

**Fig. 2:**
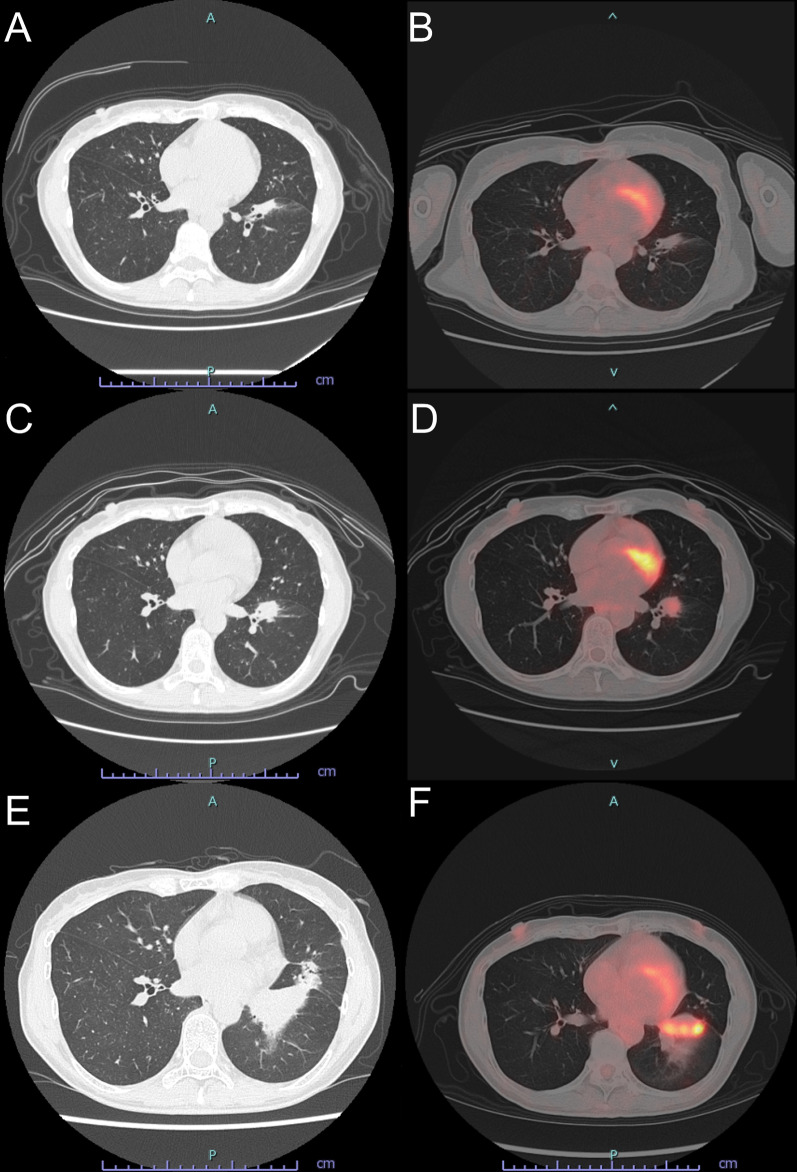
At 6 months after osimertinib administration, **A** CT imaging showed shrinkage of the primary tumor and **B** PET-CT findings indicated a low SUVmax level of 1.5. After 1.5 years, **C** the primary tumor showed regrowth and **D** SUVmax was increased to 4.55, thus SRT was performed. At 3.5 years after SRT, **E** the primary tumor showed continued growth and **F** SUVmax was increased to 16.78, which led to reference to our hospital

Afatinib administration (40 mg/day) was started, which led to partial response. At 1.5 years after starting afatinib, regrowth of only the primary tumor was noted. A re-biopsy bronchoscopy procedure was performed and acquisition of a T790M point mutation was revealed. Afatinib was switched to osimertinib (80 mg/day), resulting in shrinkage of the primary tumor shown by CT and a low accumulation (SUVmax 1.5) noted in PET-CT findings (Fig. 2A, B). However, 1.5 years later, imaging showed re-growth of the primary site and SUVmax was increased to 4.55 (Fig. 2C, D). Stereotactic radiation therapy (SRT) (52 Gy/4 fractions) was performed to treat the primary tumor.

**Fig. 3:**
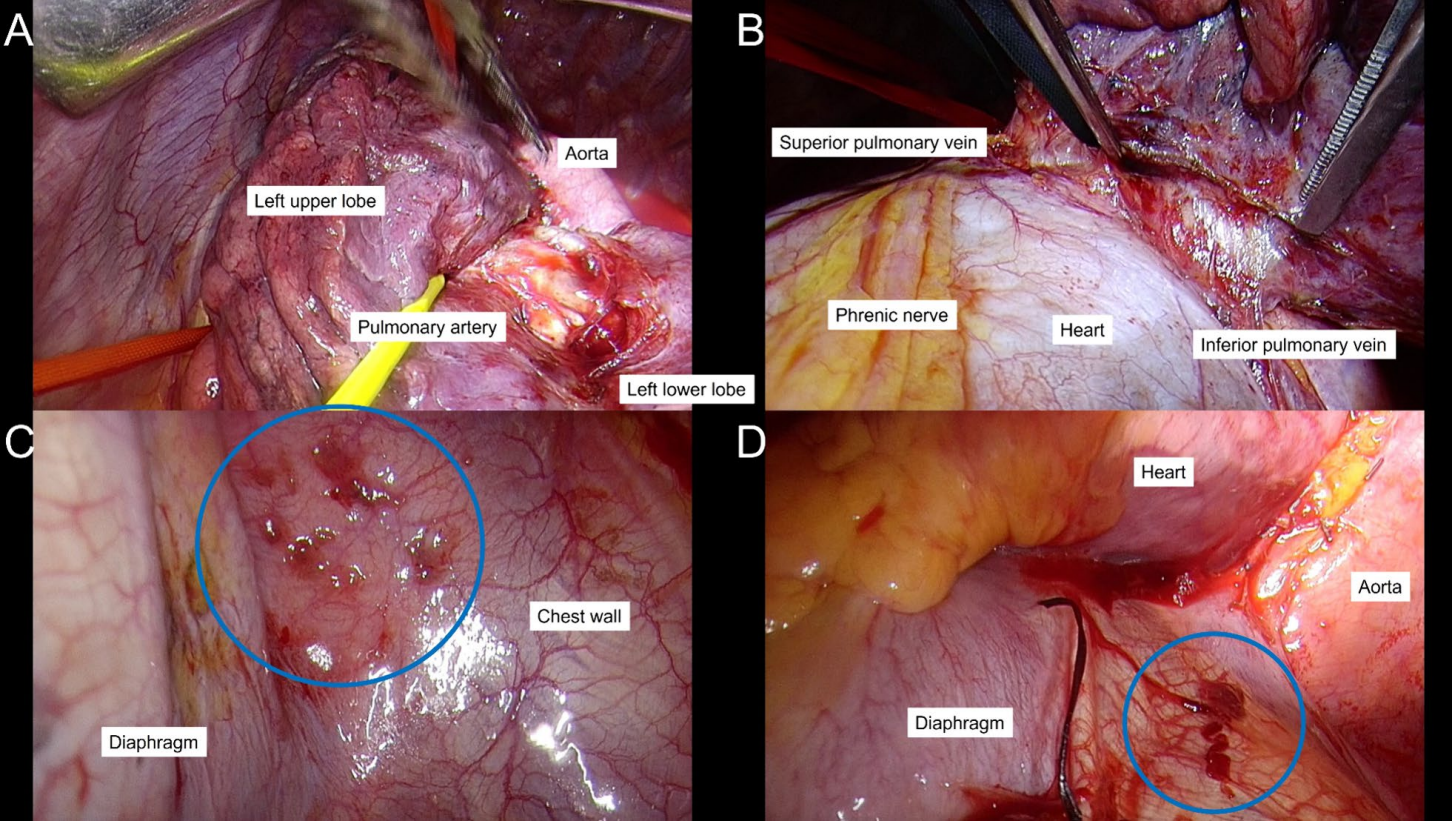
The **A** left main pulmonary artery, and **B** left superior and inferior pulmonary veins were encircled for safety because of rigid fibrosis surrounding the hilar area

At 3.5 years after osimertinib administration, all lymph nodes and distant metastases, excluding the primary tumor (SUVmax 16.78), were controlled (Fig. 2E, F), and the patient was referred to our hospital for salvage surgery. Osimertinib was discontinued 1 week before surgery, then an open thoracotomy through the 5th intercostal space was performed. Rigid fibrosis was found surrounding the hilar area, thus the left main pulmonary artery, and left superior and inferior pulmonary veins were encircled for safety (Fig. 3A, B). Although the surgical procedure was difficult because of prior osimertinib and radiation therapy, exposure and separation of the pulmonary vessels and bronchus of the left lower lobe were obtained. Intraoperative findings showed that the lingular segment of left lung had been damaged by SRT, which led to a left lingular segmentectomy with the left lower lobectomy. A mediastinal lymph node dissection was thus conducted and no lymph node metastasis was detected during the operation. Some small nodules on the chest wall and diaphragm were found, and a pleural biopsy was also performed (Fig. 3C, D). The operation was safely completed, followed by an uneventful postoperative course.

**Fig. 4:**
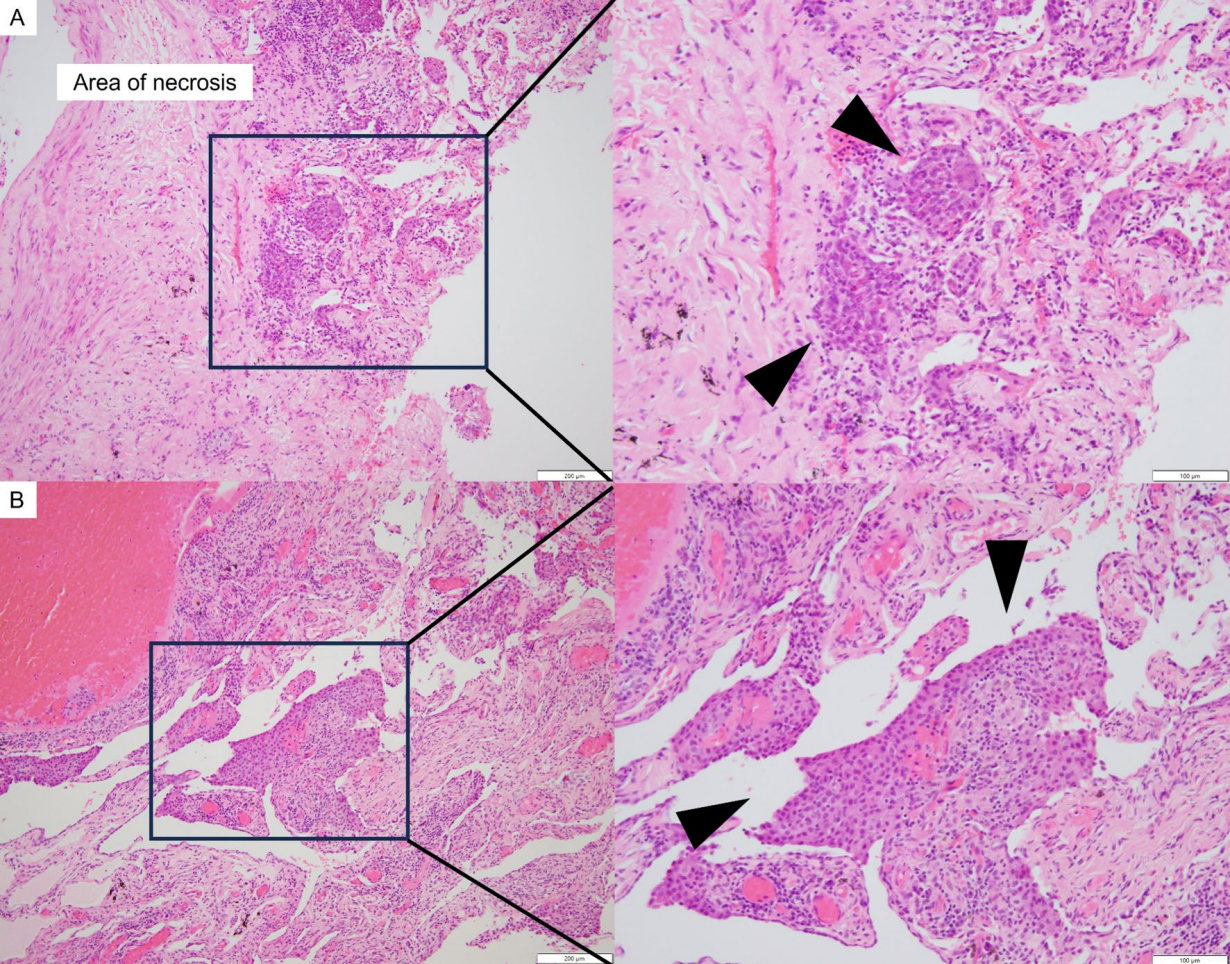
**A** Pathological findings revealed a major pathologic response by the primary adenocarcinoma, with few viable tumor cells found (arrowheads). **B** Findings of the pleural biopsy specimen confirmed pleural dissemination of the adenocarcinoma (arrowheads)

**Fig. 5:**
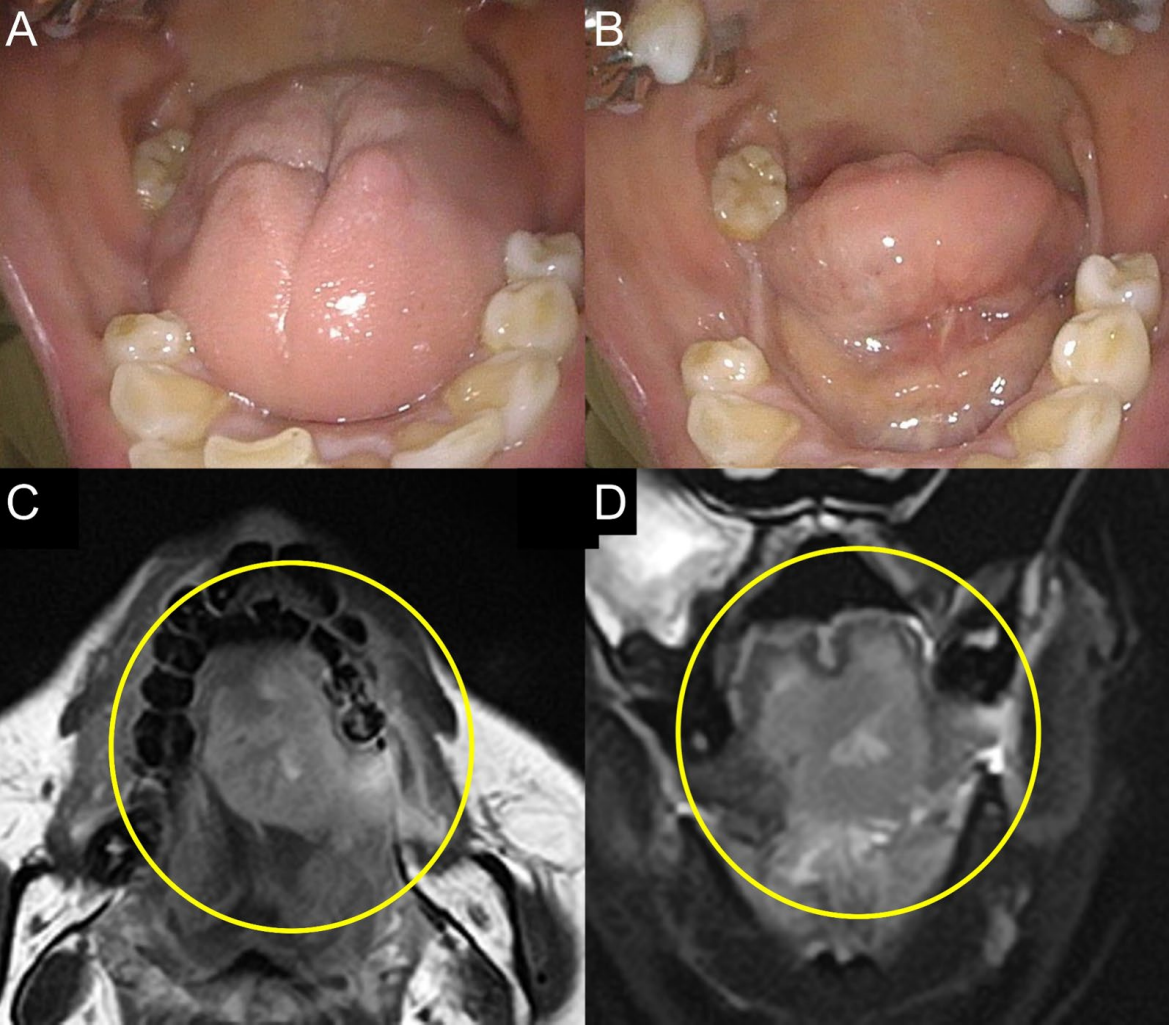
**A**, **B** The tongue showed no abnormal appearance. **C**, **D** Head MRI findings revealed tongue metastasis

The pathological diagnosis of the surgical specimen was adenocarcinoma residue (85 × 40 × 27 mm) without lymph node metastasis, though with pleural dissemination (ypT4N0M1a, stage IVA; TNM staging 8th edition) (Fig. 4A, B). The patient was discharged on postoperative day (POD) 13. On POD 16, she complained of glossodynia, though plain brain MRI findings showed no metastasis. Osimertinib was re-administered on POD 19 and glossodynia improved. Although a dental examination performed on POD 25 found no abnormal appearance (Fig. 5A, B), a head MRI examination revealed tongue metastasis (Fig. 5C, D). With continuation of osimertinib, PET-CT findings obtained 8 months after surgery revealed tongue and right adrenal gland metastases, and radiation therapy was performed for those at 11 and 13 months, respectively. At 1 year after salvage surgery, the patient was alive without disease progression after the radiation therapy under the administration of osimertinib.

## Discussion

Selection of additional therapy for a progressive lesion following TKI treatment for clinical stage IV lung cancer is difficult. We performed salvage surgery for a growing primary tumor in the present patient, who showed well-controlled distant metastases. However, the postoperative course was not optimum, as multiple distant metastases appeared, indicating limitations of local control.

Although sufficient evidence regarding the effects of salvage surgery after TKI treatment is lacking, reports of favorable surgical outcomes have been presented by Ohtaki et al. [6] and Lin et al. [7]. Nevertheless, prognostic factors related to salvage surgery for TKI-treated patients remain controversial. Several studies have discussed indications for salvage surgery in advanced lung cancer cases after TKI treatment. In an EGFR-TKI study, Ning et al. noted that locally advanced patients had better outcomes than lymph metastasis patients [8]. The report by Ohtaki et al. noted that distant metastasis did not have effects on PFS or overall survival (OS), while Chen et al. reported a trend of worse PFS for extrathoracic metastasis cases without a significant difference regarding OS [9]. Furthermore, Hishida et al. noted that TKI is a cytostatic rather than cytotoxic agent, thus its administration does not eliminate systemic micrometastatic tumor cells even in cases in which distant metastasis has disappeared [10]. In the present case, the patient initially had multiple lymph node metastases and liver metastasis, indicating that tumor cells had already spread throughout the whole body. This was implied by the fact that the primary tumor and the tongue metastasis showed heterogeneity characteristics about TKI response, in other words, tumor cells had already metastasized to the tongue before they acquired TKI resistance. Considering the cytostatic nature of TKI, resection of the primary site was unlikely to achieve disease-free status. Hence, local control such as salvage surgery or radiation therapy was performed for uncontrollable lesions by TKI in the present case. In this regard, the indication for salvage surgery should be considered because of potential micrometastasis throughout the body. Therefore, perioperative TKI withdrawal may have affected early recurrence more than timing of salvage surgery in the present case.

Observations of disease flare after discontinuation of erlotinib or gefitinib have been reported [11]. Although whether discontinuation of osimertinib can cause disease flare has yet to be fully elucidated, two such cases have been reported [12, 13]. In the present patient, perioperative discontinuation of osimertinib might have led to glossodynia, resulting in tongue metastasis, while the tongue pain symptom disappeared after restarting osimertinib administration. Attending physicians should be aware of the possibility of disease flare, even shortly following cessation of TKI. Not only tongue metastasis, but also adrenal metastasis was observed in the present case. Postoperative systemic therapy was necessary, thus it is possible that salvage surgery, which led to performance status deterioration, should have been avoided.

Tongue metastasis in lung cancer cases is considered to be rare. However, Irani reported that lung cancer was the second most common cause of metastatic tongue malignancy in their cases [14], and also noted that the prognosis of affected patients was poor despite administration of chemotherapy or radiation therapy. In the present case, tongue metastasis could not be immediately diagnosed due to a lack of gross findings and the brain MRI examination did not include the tongue. The possibility of tongue metastasis should be considered in advanced lung cancer patients with tongue symptoms.

## Conclusions

The present patient with distant metastasis well-controlled by TKI experienced consecutive systemic recurrence episodes following salvage surgery, which indicates the importance of continuation of systemic therapy for cases of distant metastasis. It is considered that these clinical findings associated with salvage surgery for advanced lung cancer will be useful for establishing an optimal treatment strategy.

## Data Availability

The authors declare that all data within this article are available upon reasonable request.

## Abbreviations

EGFR: Epidermal growth factor receptor
TKI: Tyrosine kinase inhibitor
PFS: Progression-free survival
CT: Computed tomography
PET-CT: Positron emission tomography-computed tomography
SUVmax: Maximum standard uptake value
MRI: Magnetic resonance imaging
SRT: Stereotactic radiation therapy
POD: Postoperative day
OS: Overall survival
